# An Evaluation of Radarsat-1 and ASTER Data for Mapping *Veredas* (Palm Swamps)

**DOI:** 10.3390/s8096055

**Published:** 2008-09-26

**Authors:** Philippe Maillard, Thiago Alencar-Silva, David A. Clausi

**Affiliations:** 1 Departamento de Cartografia, Universidade Federal de Minas Gerais - UFMG, Belo Horizonte, Minas Gerais, Brazil, 31270-901; E-Mail: thiago-alencar@uol.com.br; 2 Systems Design Engineering, University of Waterloo, Waterloo, Ontario, Canada, N2L 3G1; E-Mail: dclausi@engmail.uwaterloo.ca

**Keywords:** Radarsat, Unsupervised Classification, Markov Random Fields, Wetlands, Palm swamps, ASTER, Supervised Classification, Vegetation types

## Abstract

*Veredas* (palm swamps) are wetland complexes associated with the Brazilian savanna (*cerrado*) that often represent the only available source of water for the ecosystem during the dry months. Their extent and condition are mainly unknown and their cartography is an essential issue for their protection. This research article evaluates some of the fine resolution satellite data both in the radar (Radarsat-1) and optical domain (ASTER) for the delineation and characterization of *veredas*. Two separate approaches are evaluated. First, given the known potential of Radarsat-1 images for wetland inventories, the automatic delineation of *veredas* is tested using only Radarsat-1 data and a Markov random fields region-based segmentation. In this case, to increase performance, processing is limited to a buffer zone around the river network. Then, characterization of their type is attempted using traditional classification methods of ASTER optical data combined with Radarsat-1 data. The automatic classification of Radarsat data yielded results with an overall accuracy between 62 and 69%, that proved reliable enough for delineating wide and very humid *veredas*. Scenes from the wet season and with a smaller angle of incidence systematically yielded better results. For the classification of the main vegetation types, better results (overall success of 78.8%) were obtained by using only the visible and near infrared (VNIR) bands of the ASTER image. Radarsat data did not bring any improvement to these classification results. In fact, when using solely the Radarsat data from two different angle of incidence and two different dates, the classification results were low (50.8%) but remained powerful for delineating the permanently moist riparian forest portion of the *veredas* with an accuracy better than 75% in most cases. These results are considered good given the width of some types often less than 50 m wide compared with the resolution of the images (12.5 - 15 m). Comparing the classification results with the Radarsat-generated delineation allows an understanding of the relation between synthetic aperture radar (SAR) backscattering and vegetation types of the *veredas*.

## 1. Introduction

As wetlands, *veredas* (palm swamps) bear an essential role in the environment by enhancing water quality, reducing flood damage, sequestrating carbon and supporting a disproportional part of the biodiversity [1–3]. Being associated with the *cerrado* (Brazilian savanna), *veredas* are also frequently the only source of perennial water supply during the dry months of the year (in the *cerrado* of Minas Gerais the dry season can last for six to eight months) [4, 5].

**Figure 1.**
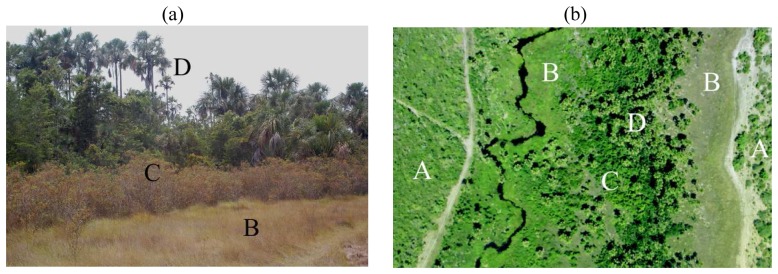
Photographs illustrating the different types of *veredas* (a) as seen from the ground and (b) from the air: A - Wooded Savanna, B - Wet meadow, C -Shrubland/Riparian forest, D – Buriti palms.

*Veredas* present varying types, ranging from wet meadows to riparian forest and are associated with the presence of Buriti palms (*Mauritia flexuosa* L.f., see Figure 1). Although their aspect can vary significantly, typical *veredas* are relatively narrow landscape features that follow a mostly intermittent stream that can either have a diffuse or well-defined channel. Their width can vary from tens of meters to a few hundred. While *cerrado* is a mostly open wooded savanna formation adapted to semiarid conditions, *veredas* are closed riparian formations characterized by hydromorphic soils with high organic content. From the contact with *cerrado* vegetation towards the lowest point of the valley, typical *veredas* show the following sequence of types: grassland (wet meadows), shrubs and trees often dominated by *Mauritia flexuosa*.

They form under very specific hydrological and geomorphological conditions; *veredas* are riparian zones of a mostly flat topography characterized by the superposition of a pervious rock layer over an impervious one causing the aquifer to surface during a sufficiently long part of the year for hydromorphic soils to form [4, 5].

The distribution of the different types are ruled by the availability of water; the central part is usually saturated all-year-round, whereas the marginal wet meadows suffer from strong fluctuations of the water table, causing the soil to be either saturated or flooded from November to March or dry from April to October [4]. As such, *veredas* can be considered, in the long term, as “barometers” of the aquifer. For example, excessive irrigation in nearby agricultural projects on very sandy soils can lower the water table and result in the irreversible degradation of *veredas*. Conversely, the construction of dams causing permanent flooding will have a similar effect causing the death of the *veredas*.

With the rapid conversion of *cerrado* to agro-pastoral activities *veredas* (*Cerrado* is the most threatened biome of Brazil with a conversion rate exceeding that of the Amazon forest [6].) are increasingly threatened. Despite being protected by the Brazilian legislation, *veredas* in numerous regions of Minas Gerais [with an area of 587.150 km2, Minas Gerais is the fourth largest state in Brazil and is dominated by the *cerrado* (≈60%), for which *veredas* represent one of its most important source of perennial water during the dry months] already show signs of advanced degradation. However, the actual degree of this degradation is still unknown as is the true extent of *veredas* in Minas Gerais [5].

### 1.1. The use of remote sensing for wetland mapping

The three primary methods of wetlands inventory are on-site field work, photo-interpretation of aerial photography and digital image processing of satellite imagery. The first two have the disadvantage of a relatively long time lag between data acquisition and map production [7]. Assuming a timely processing and interpretation, satellite remote sensing is considered the only practical method for mapping and monitoring wetlands [8]. It has been reported [9] that research is needed in the field of remote sensing to assess total wetland resource habitats, detect changes at large spatial scales and produce standardized approaches to inventory and information dissemination. For example, [10, 11] found that Landsat TM data showed a good capacity to estimate the extent of wetlands in various regions of the globe. Others have found that the presence of a dense canopy makes it difficult to separate wetlands from other forest types [12]. In the specific case of *veredas*, [13] have found that they often cannot be separated from other riparian formation not classified as wetlands. Baker *et al.* [7] have attained high classification success (> 85%) using Landsat ETM+ in combination with topographic and soil data.

Optical data have the additional limitation of not permitting the acquisition of imagery under cloudy conditions or at night. In that perspective, synthetic aperture radar (SAR) offers a promising approach, since it is unaffected by clouds or illumination conditions. SAR also has the ability (conditioned by the frequency used) to penetrate the canopy and yield information about the ground. The frequencies used by radar systems, ranging roughly from 300 MHz to 30 GHz (or wavelengths of respectively 1 m to 1 cm), interact with different parts of the vegetation. While with longer wavelengths (P- and L-band) scattering and attenuation result mainly from interaction with trunks and large branches, shorter wavelengths from the K and X bands interact primarily with leaves. Mid-range wavelengths like the C and S bands interact with a combination of leaves and branches [14].

### 1.2. Interaction between radar backscatter and wetland vegetation

Reflection of radar pulses is generated by a discontinuity in the dielectric constant of the surface, the larger the difference, the larger the reflection. The roughness at the wavelength scale (a few centimeters) being a function of the frequency, and orientation of the surface govern the amount of energy scattered back to the side-looking antenna. A water surface has a high dielectric constant, but if it has a smooth surface, it will reflect all the energy away from the antenna. Conversely, dry soil and dry bark of trees have a relatively low dielectric constant and most of the energy is transmitted or absorbed leaving a backscatter of relatively low magnitude [15]. Regardless, since energy is scattered in all directions, a significant portion is still returned towards the antenna. For soil and vegetation, it is primarily the moisture content that increases the dielectric constant and provide high radar pulse reflection [16]. Combined with a rough surface having many vertical structures, high dielectric constant of humid surfaces provide strong signal return to the radar antenna which translates into bright features on the radar image.

**Figure 2.**
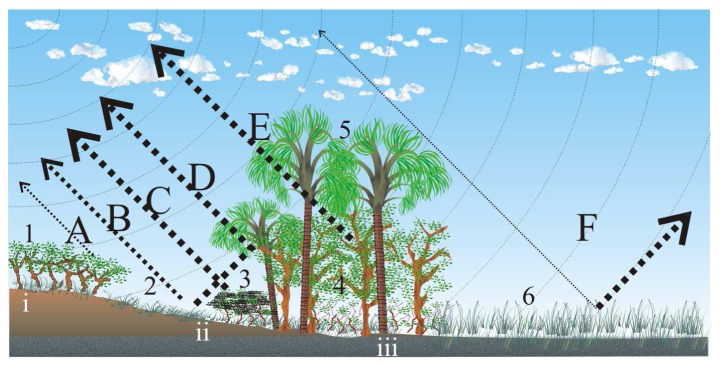
The main mechanisms governing the interaction between SAR C-band signal and a *vereda*. Legend: 1) wooded savanna, 2) intermittently dry grasses, 3) shrub, 4) trees with a predominance of 5) *Mauritia flexuosa* L.f. and 6) permanently wet grasses, i) dry sandy soil, ii) hydromorphic, almost permanently moist soils, iii) the aquifer is permanently close to the surface. In A) the dry wooded savanna and dry soils have a low dielectric constant but a high scattering potential. In B) the grasses have a relatively high scattering power and can have a high dielectric constant when wet or when the soil is saturated. In C) and E) the shrub and trees growing on hydromorpic soils are good scatterers and are moist with a high dielectric constant. In D) the riparian forest is a good scatterers with a high dielectric constant and can act as a corner reflector on its margin. Finally, in F) flooded grasses cause most of the signal to be reflected away from the SAR antenna.

The fact that radar backscattering is very sensitive to moisture is a very useful characteristic for wetland mapping. With some exceptions like mud flats, humid or saturated soil of wetlands are usually well vegetated and have a relatively rough surface that will strongly scatter the microwave pulses in all directions yielding a relatively high signal return [17]. A third SAR phenomenon is frequently observed in wetland environments when a vegetated surface is flooded; the pulses are first reflected away by the smooth flooded ground then back to the antenna by the perpendicular vegetation structure. The combination of the two perpendicular surfaces act as a corner reflector that causes the radar pulses to bounce back at the antenna, a phenomenon called double bounce [8, 18]. This is especially true for longer wavelengths (L- and P-bands). In the case of shorter wavelength like C-band (5,3 GHz or λ= 5,6 cm) the increase of backscattering appears to be primarily due to increased moisture combined with a rough canopy structure. Conversely, in wetlands with no woody plants, the increase in specular scattering caused by standing water tends to decrease radar backscattering [14]. This situation is likely to happen in the grassland that margin the *veredas* during the high wet season when they are flooded. These mechanisms are illustrated and explained in Figure 2.

SAR data has proven its utility for mapping wetland and flooded forests extents [19], but its ability to discriminate between vegetation type and form is mainly dependent on the use of multi-date, multifrequency or multi-polarization radar data or a combination of these multiple data sets [14]. Studies have shown that using Radarsat-1 in combination with optical Landsat data can significantly improve land cover classification [20–22]. However, it should be mentioned that these studies were aimed at improving land-cover mapping from Landsat data with a 30 m resolution cell and that radar derivative (mostly texture features) were used. Generally speaking, unless one would want to separate very different features (wetland vs. forest vs. urban), optical remote sensing is still preferable to single-date/frequency/polarization radar data for such applications as classification of vegetation types and, only when integrated with optical data has a single SAR image been found to bring a significant improvement to the classification of vegetation [23].

### 1.3. Objectives and organization of the article

The objective of this research was to evaluate the potential of Radarsat-1 (SAR) and *Advanced Spacebone Thermal Emission an Reflection Radiometer* or ASTER (optical) satellite data for delineating and characterizing *veredas* at the 10×10 - 30×30 m scale (A range of resolution cells, generally considered as “fine” [8] but not “high”.). Radarsat-1 was the SAR of choice for its recognized potential for mapping wetlands and flooded forests [14, 19, 24–27]. ASTER optical data from the Terra satellite were selected as an improved substitute to Landsat [28] in terms of spatial resolution.

Unless strongly filtered or when texture derivatives are used, traditional pixel-based classification algorithms do not perform well with single-date/frequency/polarization SAR data [20, 29]. MRF-based (Markov random field) classification algorithms have yielded good results in other SAR image classification applications [30, 31]. The algorithm developed by [31] has successfully been applied by the authors in a sea ice classification application [32] and has yielded promising results for palm swamp delineation [33]. The same algorithm is being tested here for the unsupervised delineation of *veredas* using the hydrographic network as a starting point. Because *veredas* are always associated with the hydrographic network, this knowledge has been used to limit processing to a buffer zone on either side of the streams. It is hypothesized that this measure contributes to reduce class confusion and to increase classification success rate.

Conversely, classical pixel-based classification algorithms usually work well with optical data or combinations of optical and SAR data (or derivatives) in the selected range of spatial resolution. Different combinations of ASTER bands and Radarsat-1 images were evaluated for the supervised classification of the different types encountered in typical *veredas* as a means of characterization.

Finally, the results from the SAR and optical data are compared by spatially intersecting their results to assess the potential of Radarsat data and the unsupervised MRF-based classification approach for delineating *veredas*. This approach helped understand what types were actually captured by the SAR data and the MRF-based unsupervised classification. The use of two different angles of incidence at two different phenological times brings further insight on the optimal SAR C-band image parameters.

## 2. Materials and Methods

### 2.1. Study Area

**Figure 3.**
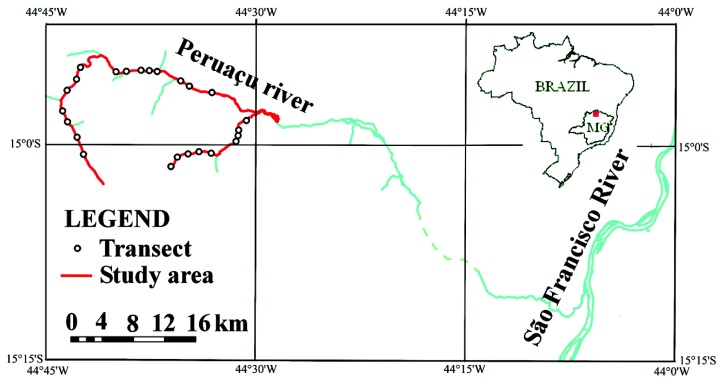
Location of the study area.

The study area is situated along the course of the Peruaçu river in Northern Minas Gerais - Brazil, in a region called Chapadão das Gerais. The area was chosen for being one of the rare well preserved regions of the Brazilian savanna having ideal environmental conditions for the research (being a protected area) and having some of the longest *veredas* areas of the country (Figure 3). The study area covers about 540 km^2^ and about 50 linear km of *veredas*. It also completely encloses the *Veredas* do Peruaçu State Park (≈ 31 km^2^). The Peruaçu river is a tributary of the São Francisco river, the third largest watershed of Brazil. The region's bedrock is composed of a layer of sandstone (usually highly weathered) over carbonates from the superior Proterozoic. The topography is mostly flat and slopes are very smooth. The climate is semiarid, with an average temperature of over 25°C. Precipitation averages 124 mm per month between October and April and less than 2 mm between May and September [34].

### 2.2. Imagery

Four Radarsat-1 images were acquired for the present study from a “Data for Research Use” (DRU) project promoted by the Canadian Space Agency (© CSA - http://www.space/gc/ca). The images were acquired in the “standard beam” mode for two distinct periods corresponding to the two main phenological seasons; April or the end of the wet season when the soil is saturated and September or the end of the dry season when the hydrological balance is at its lowest. For each period, two images of different incidence angles (the angle of incidence is the angle between the line of sight from the radar to the ground and the vertical direction) were acquired: S2 with 24° to 31° for the near and far range respectively and S6 (41° to 46°). The image pixels have a spatial resolution of 12.5×12.5 m and a radiometric depth of 16 bits. SAR images with these characteristics were ordered in an effort to assess the best period/incident angle for delineating the *veredas*. It was expected that S6 scenes would be more affected by volume scattering and vegetation structure consequently [35, 36] and that the S2 scenes would respond more to direct scattering and soil moisture.

One ASTER image from the Terra/EOS AM-1 satellite (http://terra.nasa.gov) of the study area was acquired on 21 August 2006. The ASTER instrument is composed of three subsystems: VNIR (visual and near infrared) with three bands (green 0.52-0.60 μm, red 0.63-0.69 μm and infrared 0.76-0.86 μm) and 15 m ground resolution, SWIR (shortwave infrared ranging from 1.6 μm to 2.43 μm) and 30 m ground resolution and TIR (thermal infrared) with a resolution of 90 m. To take full advantage of the fine ground resolution of the VNIR and SWIR subsystems, bands from the TIR were not used in this study. Even though TIR could prove useful since *veredas* are expected to be cooler than the surrounding savanna, the 90 m resolution was considered too broad for the application at hand. Some of the SWIR bands (2, 3 and 5) were included in addition to the three VNIR bands.

All four SAR images and the ASTER image were geometrically corrected using *in situ* ground control points collected with a navigation GPS and image-to-image based on a geo-referenced Landsat ETM+ panchromatic band with a ground resolution of 15 m. Since the relief is mostly flat, displacement effects caused by the incidence angle were almost negligible and all five images had mean square errors below 20 m. Speckle noise was not filtered from the SAR images because speckle noise algorithms tend to erode class boundaries when unsupervised segmentation is applied, reducing the quality of the classification [31]. A standard radiometric correction was applied to the three VNIR bands of the ASTER image [37].

### 2.3. Field Work and Ground Truth Data

**Figure 4.**
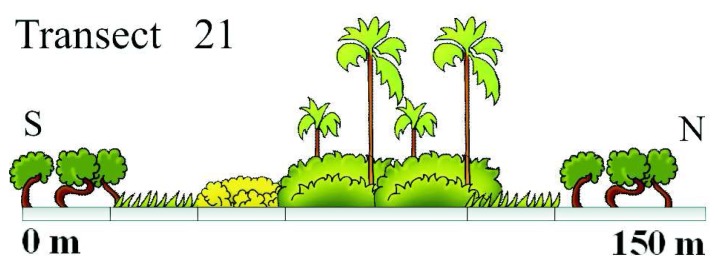
Example of graphically enhanced transect across a narrow stretch of *vereda*. Note the *buriti* palms in the center, bordered by shrub, grasses and wooded savanna successively.

*Veredas* are by nature difficult to access by land. Except for the usual fringe of grass on both sides, the center is either flooded or saturated and the vegetation is very dense. Our field campaign consisted of a series of 24 transects made at intervals being multiples of 500 meters approximately. The length of the transects ranges from 142 m to 598 m with an average of 299 m. Each transect was started and ended with 40 to 50 m in the savanna type. The *vereda* was crossed by foot and every change of type in the vegetation was recorded and positioned using a navigation global positioning system (GPS). Despite the imprecision of the navigation GPS estimated at about 10 m, all transect were geometrically consistent, in terms of alignment, and a good visual match was obtained with both SAR and optical imagery. These transects were plotted and incremented with vegetation artwork as showed in Figure 4. The transects were rasterized to match the size of the resolution cells of both image types (Radarsat-1 at 12.5 m and ASTER at 15 m). Other more traditional (small areas) training/validation sites were also collected in locations of easier access such as in the savanna and sandy areas.

Two sets of reference data were created from these transects. In the first one, the different types were reclassified in two classes: 1) vereda and 2) non-vereda to assess the success rate of the classification of the Radarsat images in delineating the extent of the *veredas*. An alternate set was created where the meadow (grass) type was removed from the vereda class and added to the non-vereda class since it was not clear, from visual interpretation of the Radarsat images, in which class it would fall. For the second set, the original reference vertices were converted into reference pixels of 15 meters for the classification of the ASTER image into the different types (see Table 1 for their description). In both cases, pixels located at transition points (between different types for ASTER or between vereda and non-vereda for Radarsat) were eliminated to keep only the pixel unequivocally containing a single vegetation class. Half of these reference pixels were randomly selected for training while the other half was reserved to validate the classification results. The different classes recorded are described in Table 1.

Botanical material was also collected for identification and samples of soil were hermetically bagged for soil moisture to be measured in laboratory. The soil moisture data was not collected at the same time as the image acquisition but at the beginning of the dry season and at least two weeks after the last precipitation event. Although not coincident in time, these data are still helpful to understand the difference between the different vegetation types and their respective soil.

### 2.4. Hydrography-based buffering

Knowing that *veredas* are always found along streams (most authors consider that *veredas* necessarily follow the natural hydrographic network), the hydrographic network was used to create a buffer zone to limit the regions used for processing. *Veredas* are rarely wider than about 500 meters so a 2,000 meter-wide corridor can easily account for all imprecisions in the hydrographic network of 1:100,000 topographic maps and include the widest of *vereda*. Medium scale topographic maps are available in digital form for most of Brazil and although most of them are outdated (> 25 years old), dislocation errors are likely to fall well within the 1,000 m on both side of the streams. Such process was done using the buffering tool of a standard geographic information system (GIS) to create a mask that was later applied to all Radarsat image data.

### 2.5. Delineating the veredas using the Radarsat Images

Classification of the Radarsat images was performed in two steps: 1) unsupervised classification and 2) labeling. The unsupervised classification was done using the MAMSEG (Modified Adaptative Markov random fields SEGmentation) algorithm, developed by [31] and based on Markov random fields (MRF). The advantage of MRF models lies in their inherent ability to describe simultaneously the local spatial context (the relationship between neighboring pixels) and the feature characteristics of each segment (from the distribution of spectral values for example). This is most appropriate since pixels of a satellite image cannot be considered independent processes but are spatially correlated. The MAMSEG algorithm has proven to be a powerful classification tool for both artificially textured images and SAR sea ice images [31, 32].

The MRF model [38] assumes that the conditional probability of a pixel given its neighbors is equal to the conditional probability of that pixel given the rest of the image. This makes it possible to consider every pixel within its neighborhood as an independent process facilitating its mathematicalmodeling [39]. Within a Bayes rule framework, the conditional probability of a pixel belonging to a given class (or segment in the unsupervised case) Y_i_ is equal to:

(1) $$\text{P}({\text{Y}}_{\text{i}}|\text{x})=\frac{\text{p}(\text{x}|{\text{Y}}_{\text{i}})\text{P}({\text{Y}}_{\text{i}})}{\sum_{\text{i}}\left[\text{p}(\text{x}|{\text{Y}}_{\text{i}})\text{P}({\text{Y}}_{\text{i}})\right]}$$

P where ***p***(***x*** |***Y_i_***) is the conditional distribution of vector *x* given class/segment ***Y****_i_* and ***P***(***Y****_i_*) is the prior probability of the ***Y****_i_* class. Suppose that the energy associated to the prior probability is E_r_ and that E_f_ represents the energy of the spatial context ***p***(***x***|***Y****_i_*), then the general energy formula is given by [40]:

(2) $$\text{E}={\text{E}}_{r}+\alpha {\text{E}}_{f}$$

where E_f_ is the energy form of feature vector *f* having k dimensions. Assuming a Gaussian distribution E_f_ can be modeled as:

(3) $${\text{E}}_{\text{f}}=\sum_{\text{s},\text{m}={\text{Y}}_{\text{s}}}\;\left\{\sum_{\text{k}=1}^{\text{k}}\left[\frac{{\left({\text{f}}_{\text{s}}^{\text{k}}-\mu_{\text{m}}^{\text{k}}\right)}^{2}}{2{\left(\sigma_{\text{m}}^{\text{k}}\right)}^{2}}+\log\left(\sqrt{2\pi \sigma_{\text{m}}^{\text{k}}}\right)\right]\right\}$$

where μ_m_ and σ_m_ are the mean and standard deviation of *m*th class in the *k*th feature vector. E_r_ represents the energy of the labels (classes) in the neighborhood of the pixel being analyzed based on a system of clique (generally pairs or triplets of contiguous pixels):

(4) $${\text{E}}_{\text{r}}=\sum_{\text{s}}\;\left[\beta \sum_{\text{t}\in {\text{N}}_{\text{s}}}\delta \left({\text{y}}_{\text{s}}\;,\;{\text{y}}_{\text{t}}\right)\right]$$

where *y_s_* and *y_t_* are the respective class of pixels *s* and *t* inside the *clique*, and δ(*y_s_; y_t_*) = -1 if *y_s_* = *y_t_* and δ(*y_s_*; *y_t_*) = 1 if *ys* ≠ *y_t_*. β is a constant. In the absence of training samples to determine the labels of the pixels of the *clique*, these are initially randomly determined and gradually stabilize by iteration.

In equation 2, σ is a parameter that sets the proportions of the relative contribution of *E_r_* and *E_f_* within *E*. The adaptation of [31] makes σ iteratively change the weighting between the spectral (global) and spatial (local) components; early iterations favor the spectral component and increased iterations gradually increase the weight on the spatial component.

Three parameters need to be specified for the classification to take place: 1) the number of classes, 2) the number of iterations and 3) a mask to limit the classification (this can ultimately be the whole image). In our case, the classification was binary (“*vereda*” and “non-*vereda*”) and the number of iterations varied between 50 and 120 with increments of ten. The mask consisted of a buffer of 1000 m on both side of the hydrographic network to include all the *veredas* which are no larger than about 500 m in the region. Eighty iterations were sufficient for the result to converge and this number of iterations was used in all tests.

### 2.6. Classification of the veredas type

An image dataset was created by joining the three ASTER VNIR bands, three of the six SWIR bands (bands 2, 3 and 5 had the least visually noticeable noise) and the four Radarsat-1 scenes that were previously filtered using a 3×3 median filter to reduce speckle noise. The set was given a standard resolution cell of 15× 15. Pixels of the SWIR bands were simply duplicated while the Radarsat-1 scenes were resampled by bilinear interpolation. In many cases we observed in the field that the different vegetation types were almost systematically arranged in parallel strips along the hydrographic network but that their width was often smaller than 20 meters. To overcome the mismatch between image resolution (15 m) and target width, some classes with similar characteristics had to be merged. The resulting classes were: 1) dense wooded savanna, 2) sparse wooded savanna, 3) grass (or meadow), 4) shrub, 5) riparian forest, 6) sandy soil with little vegetation and 7) open water.

Classification algorithms can be grouped into parametric and non-parametric. The latter have the advantage of not assuming a distribution function (e.g. Gaussian or Gibbs) and are less restrictive regarding the number and location of samples for statistical training. Both the Fisher criteria and the Mahanalobis distance were tested. The Fisher linear criteria classifier uses the training data to construct a linear function that combines all the features (or bands) to maximize the variance between classes and minimize the variance within class [41].The Mahanalobis distance [42] is used to measure the distance between a single observation **x** and a class distribution (μ; Σ). Although it uses the mean and covariance matrix, unlike the maximum likelihood it does not assume a Gaussian distribution.

Both the Fisher criteria and the Mahanalobis distance were tested with and without first applying a segmentation routine; an approach called ECHO (Extraction and Classification of Homogeneous Objects) [43]. The classification tests presented here were produced with the Purdue/NASA MultiSpec software package. The MultiSpec classification scheme can classify any combination of the training areas, testing areas and/or the entire image and reports the results with a wide range of statistics. [43] give a complete description of the MultiSpec package. A separate set of ground truth data that was not used for training the algorithm, was randomly selected to test the accuracy of the classification results.

### 2.7. Statistical inference

Because the Kappa statistic has been known to overestimate the degree of chance agreement [44] and has generated a fair amount of controversy, it was not used for comparing the results. Instead, the McNemar test was used to compare the results since the same samples were used for all classification tests and were not therefore independent as would require a Kappa difference test [45]. The McNemar test computes a *Z* statistic from a two by two matrix based on correctly and incorrectly classified pixels in both classifications as follows:

(5) $$\text{Z}=\frac{\text{f}12-\text{f}21}{\sqrt{\text{f}12+\text{f}21}}$$

where *f*12 represent the pixels that are correctly classified in the first classification and incorrectly classified in the second classification and *f*21 represents the opposite situation. *Z* values of 1.96 and 2.58 were considered for the 95% and 99% levels of confidence respectively.

## 3. Results and Discussion

### 3.1. Delineating veredas

The four Radarsat-1 images were successfully segmented and labeled. Because of its higher dielectric constant and high scattering capacity, a *vereda* tend to be brighter than its surrounding. The soil moisture is considered a significant component responsible for the increase in the dielectric constant. Table 2 shows the gravimetric moisture measured in the soil of the main vegetation types during the dry season.

**Figure 5.**
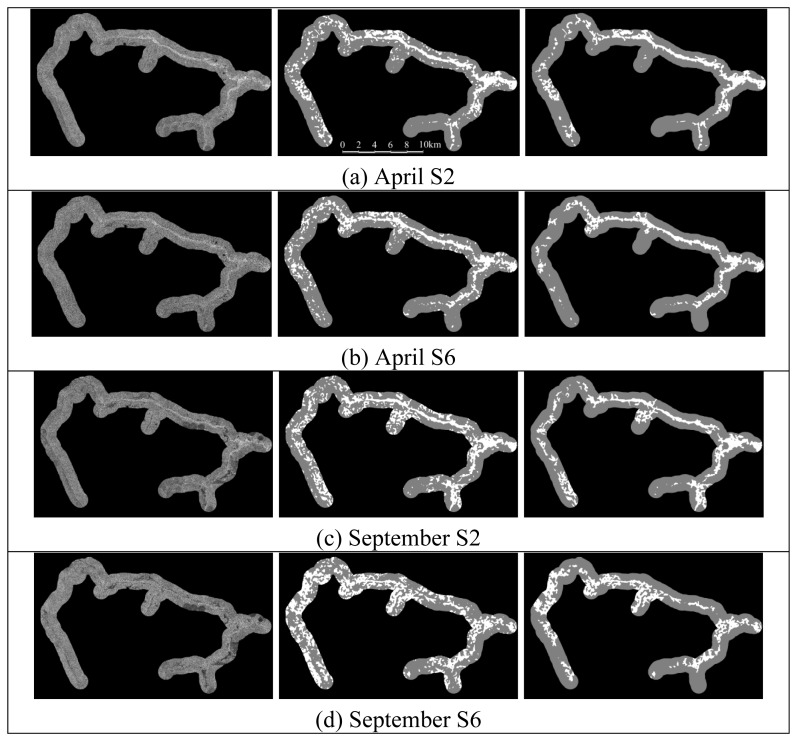
SAR images (left), classification results obtained with the MAMSEG algorithm for the two periods and the two incidence angles using 80 iterations (center) and classification results after they have been “cleaned” using the stream network and the contamination algorithm (right).

The moisture gradient is quite neatly expressed especially for the riparian forest (soil samples from the shrub type were excluded because only two valid samples were processed and results were inconclusive). For this reason, the class with the highest backscattering average was labeled as “*vereda*” leaving the remaining class as “non-*vereda*”. Only the segments directly connected to the hydrographic network were kept using a “contamination” process; the pixels belonging to both the *vereda* segments and the stream were marked and then all (four-) connected pixels to these also received an approval mark. The remaining pixels were attributed the non-*vereda* class. This process made sure that other regions such as swampy pools were not wrongly classified as *vereda*. Figure 5 shows the classification results for the four Radarsat-1 images. The first observation is that despite a relatively low visual contrast and a significant presence of noise, the MAMSEG algorithm did extract strips of higher backscattering, perhaps even better than the naked eye could have done. Eliminating the non-connected segments further improve the visual results. Before considering ground truth and the contingency table, a number of visual observations are inferred below.

- Eliminating unconnected pixels from the results using the hydrographic network and the “contamination” approach is a necessary step.
- The April (end of the rain season) images offer better visual contrast and visually more consistent results.
- The lower incidence angle (S2), prioritizing direct backscattering (as opposed to volumetric) tends to produce more consistent results in terms of continuity (less gaps) and width of the *veredas*. Because *veredas* vary in width and many sections are quite narrow, the volumetric backscatter does not always produce a significant contrast with the surrounding savanna vegetation.
- *Veredas* near the headwaters are more difficult to detect probably because of their narrower width and less saturated soils. Soils in the headwater *veredas* were found to be generally dryer therefore the dielectric constant should be lower.
- Combining higher incidence angle (S6) and dry season (September) produce the worse visual results. This observation supports the second and third statement and *veredas* are much harder to detect (even visually) in the September S6 image.

The difference in moisture suggests that, in terms of soil, grassland is more likely to be associated to the savanna than it is to the riparian forest. For this reason, two versions of the confusion matrix were built (Tables 3 and 4).

In Table 3, the wet meadow (grasses) type has been kept as part of the *vereda* class since, strictly speaking, this strata is part of the *vereda* complex. However, because it was not clear if the grassland would be captured as being part of the strip with higher backscatter response, a second version of the contingency matrix was made excluding it from the *vereda* class and incorporating it as non-*vereda* (Table 4). The wet meadow type usually occupies the transition zone between the (dry) savanna and the (wet) palm swamp so that its state changes from saturated to dry during the year. Since April represents the end of the wet season, the soil is not completely saturated and its dielectric constant is probably less than at high rain season.

Table 3 confirms the observation made above that the combination of “wet” season image and the low incidence angle shows better potential for delineating the *vereda* complex but the difference of 1.95% to the second best (September S2) is marginal. Like for Table 3, Table 4 confirms that the April S2 image yields the best results for delineating *veredas*. Comparing Tables 3 and 4 leads to the consistent observation that excluding the grassland leads to better results in all cases.

Table 5 shows the Z statistic computed to compare all the results of Tables 3 and 4. At a 95% level of confidence, none of the differences between results including grassland type in the *vereda* complex was found to be significant. However, if the grassland type is excluded, than the results from the April S2 image are significantly better than the results from all other images.

The same McNemar test was applied to assess if there is a significant difference between including grassland (Table 3) and excluding them (Table 4). Z statistics of 6.110, 3.976, 3.710 and 4.939 were obtained for April S2, April S6, September S2 and September S6 respectively which are all significant at 99% (Z = 2.58).

### 3.2. Classifying the different types

The combination of the Mahanalobis distance classifier and the ECHO routine produced systematically higher classification success, and since the choice of classifier is outside the scope of this article, only these results are presented here. In order to evaluate the contribution of the different ASTER bands and Radarsat scenes, a “knock-out” approach was used as a preliminary feature selection scheme [46]. The “knock-out” approach consists in progressively eliminating the least useful features by successive classifications until only one is left. Since finding the best subset of features is a combinational problem (10 features represent 1023 different combinations), the “knock-out” approach is a very effective suboptimal method for ranking the features in the image set (only 55 or [10(10+1)/2] combinations need to be tested).

The result of the “knock-out” method of feature ranking is presented in Table 6. The fact that the three SWIR bands are the least useful was partly expected since its ground resolution is the coarsest and at least three of the classes are expected to have a narrow configuration. The two April Radarsat-1 scenes (S2 and S6) come in third and fourth place above the two September scenes suggesting that April (the end of the wet season with moist vegetation and soils) is more appropriate for separating the different types. The fact that the red band (VNIR 2) is ranked lower than the two SAR (April) scenes was rather surprising but it should be noted that the difference in performance with these three features is very small (see Kappa results in Table 6).

Based on these ranking results, different classifications were tested using different subset of features from the image set and are presented in decreasing order of performance (based on the Kappa results of the validation set) in Table 7. The VNIR achieved a higher score with an overall success of 78,8% followed closely by the subset integrating the VNIR and the two Radarsat-1 scenes from April. The Radarsat-1 scenes do not bring any improvement and the results remain not significantly different. The inclusion of a SWIR band (3 which ranked best) only contributes to decrease the overall accuracy. As for the attempt of classifying the types with only the four Radarsat scenes, the overall success was only 50,8% which supports the statement made earlier that single phase/frequency SAR is usually not sufficient to discriminate between vegetation types.

**Figure 6.**
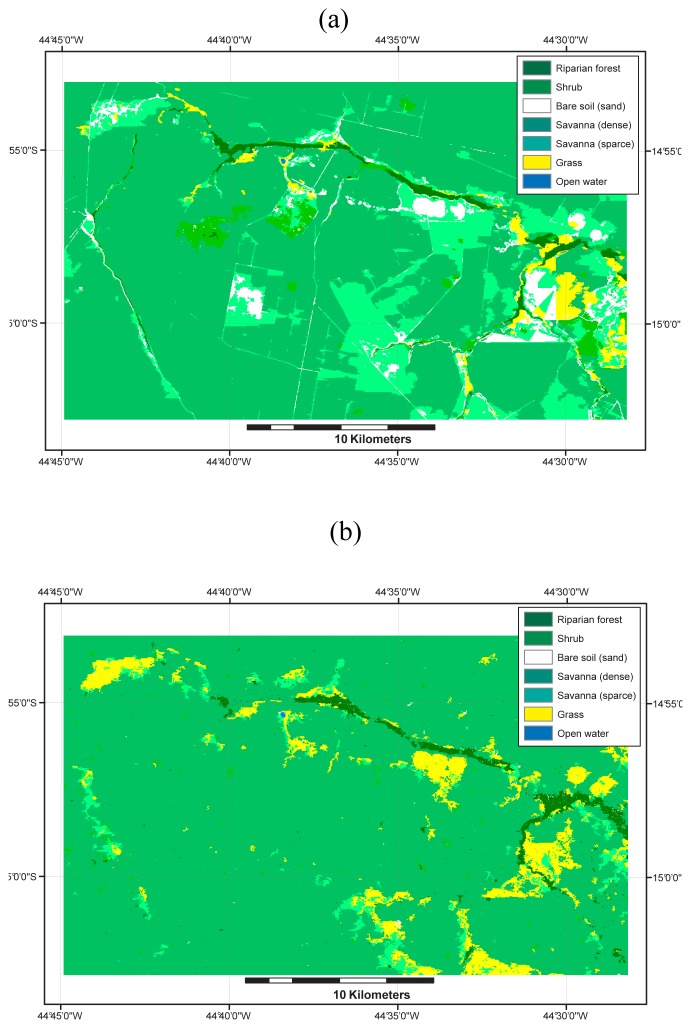
Results obtained from the classification of the vegetation types a) with the ASTER image (VNIR only) and b) with the four Radarsat-1 scenes combined.

The results of VNIR-only classification are shown in Figure 6 and in Table 7. This classification corresponds to all the classes described earlier in Table 1. The overall performance of 78.8% is good considering the marginal spatial resolution and the narrow nature of three of the classes. Most of the confusion between these classes are concentrated between shrub and grassland, shrub and riparian forest, sparse and dense savanna. These classification errors can be mostly attributed to training and test pixels with mixed vegetation cover which was often unavoidable because of the narrow nature of these vegetation types. In other words, resolution cells in the order of five to ten meters would have been preferable but might also have caused other problems such as increase data variance and texture related problems.

### 3.3. Comparing the Results

In order to assess the effectiveness of the unsupervised classification of the Radarsat-1 scenes using the MRF-based segmentation, the results were compared with the ASTER VNIR supervised classification (types). The comparison was done by intersecting both classification in the following manner:

(6) $$\text{if}\;{\text{SAR}}_{\text{class}}=\text{veredas}\;\text{then}\;{\text{ASTER}}_{\text{class}}\;\text{else null}$$

The results of these crossing operations were then compared with the classification of the different types given that the same 2,000 m buffer was applied to the classified image. Table 8 reveals that although a large proportion of the *veredas* were captured by the approach using the MAMSEG algorithm, it also captured large areas of savanna that do not belong to the *vereda* class, especially in the two September images. It is our understanding that these “artifacts” were created by the lack of clear contrast between the *veredas* and the other vegetation covers. A strong correspondance between the riparian forest type and what was classified as *vereda* in the SAR image is clearly shown for all four SAR results with an average percentage of 74.2% of the riparian forest being captured by the approach. Even when considering the accuracy of the riparian forest class is around 84%, these results can be regarded as reasonable. A visual comparison between any of the SAR results (Figure 5 and the ASTER classification in Figure 6) reveals that the headwater sections were not well recognized and are characterized by numerous gaps attributed to the narrowness of the *veredas* in these regions. These areas of *veredas* are also drier in comparison with downstream wider areas. The grassland was only partially captured confirming our initial observation that these regions might not be well captured at least in their dry portion.

## 4. Conclusions

An experimental evaluation of synthetic aperture radar (Radarsat-1) and optical (ASTER) data for delineating and characterizing *veredas* has been concluded in this paper and the results suggest that both data types show good potential providing that appropriate classification tools are used. An original unsupervised algorithm based on Markov random fields (MAMSEG) proved better than conventional pixel-based classification for delineating *veredas* using SAR data if the processing is restricted to a buffer zone following the hydrographic network.

For the SAR-based delineation, the results suggest that images acquired during or at the end of the rain season with a low incidence angle tend to yield better results. The advantage of using the hydrographic network buffering and the MAMSEG algorithm is that no ground truth data is required prior to the classification. The study also gives an insight on the mechanisms ruling the relation between the *veredas* types and SAR backscattering response.

The classification of the vegetation types revealed that the SAR-based delineation of the *veredas* left important gaps, but that the riparian forest portion was generally captured unless it lies near the headwaters (dryer soil) or has a very narrow width. The VNIR instrument of the ASTER image has a marginal ground resolution for capturing the different vegetation types of the *veredas* that are sometimes narrower than a single pixel but provided reasonable accuracy (***k̂*** ≈ 75%) for mapping the main vegetation forms.

*Veredas* are very important wetland complexes for the *cerrado* biome and various government agencies have already outlined the need for mapping their extent and state. We believe that the automatic classification of SAR images (Radarsat or other C-band SAR imagery) is an efficient means of providing a rough evaluation of their extent. Fine resolution optical data like ASTER could then be used to characterize their type. More precise solutions would almost unavoidably involve high-resolution data (e.g. Ikonos, Quickbird) at a much higher cost.

## Figures and Tables

**Table 1.** Class name and description of vegetation type of the *veredas* and surrounding areas.

**Class name**	**Description**

Grassland	A relatively narrow (<50m) band of grass usually inserted between the savanna and the shrubland or the riparian forest;
Shrub	A narrow band of shrub that may or may not be present between the meadow and the trees;
Riparian forest	A relatively dense canopy of trees with emerging *buriti* palms and having a width that can vary from less than fifty meters to a few hundreds of meters;
Sparse wooded savanna	A sparse community of small trees (<5m) and shrub with frequent patches of bare soil;
Dense wooded savanna	A dense community of small trees (<7m) and shrub with a continuous canopy;
Bare soil (sand)	Areas of very little vegetation (usually grasses and some shrub) characterized by loose sand;
Open water	Class almost exclusively represented by small lakes but might include some open water areas within the *veredas*.

**Table 2.** Summary of gravimetric soil moisture data collected in the study area.

**Vegetation type**	**Number of samples**	**Gravimetric moisture**

Wooded savanna	4	4.41%
Grassland/wet meadows	9	8.91%
Riparian forest	9	59.54%

**Table 3.** Confusion matrices and overall success for the classification of the four Radarsat-1 images into *vereda* (including grassland) and non-*vereda*.

	**Vereda pixels** (489)	**Non-vereda pixels** (589)	**Total** (1078)
**April S2**		Overall:	**64.1%**
vereda	236	134	370
non-vereda	253	455	708

**April S6**		Overall:	**61.5%**
vereda	271	197	468
non-vereda	218	392	610

**Sept. S2**		Overall:	**62.2%**
vereda	280	199	479
non-vereda	209	390	599

**Sept. S6**		Overall:	**62.0%**
vereda	178	99	277
non-vereda	311	490	801

**Table 4.** Confusion matrices and overall success for the classification of the four Radarsat-1 images into *vereda* (excluding grassland) and non-*vereda*.

	**Vereda pixels** (405)	**Non-vereda pixels** (672)	**Total** (1077)
**April S2**		Overall:	**69.2%**
vereda	222	149	371
non-vereda	183	523	706

**April S6**		Overall:	**64.7%**
vereda	248	223	471
non-vereda	157	449	606

**Sept. S2**		Overall:	**65.3%**
vereda	255	224	479
non-vereda	209	390	599

**Sept. S6**		Overall:	**66.1%**
vereda	159	119	278
non-vereda	246	553	799

**Table 5.** The McNemar test: Z statistics and significance of Radarsat image classification differences for the reference set including (left) and excluding (right) the grassland type (values in bold are significant at 95%, Z=1.96).

	**Grassland included**			**Grassland excluded**		
	April S6	Sept. S2	Sept. S6	April S6	Sept. S2	Sept. S6
April S2	1.1826	0.8705	1.5416	**2.3570**	**2.3438**	**2.2156**
April S6	-	0.4048	0.2941	-	0.2882	0.2349
Sept. S2	-	-	0.7047	-	-	0.0586

**Table 6.** Feature ranking results obtained from the “knock-out” approach.

**Rank**	**Feature**	**Kappa** (***k̂***)	**Feature used** (by rank)
1 (most useful)	VNIR 1 (green)	61,1%	1
2	VNIR 3 (near infrared)	68,9%	1 2
3	SAR April S2	71,0%	1 2 3
4	SAR April S6	72,2%	1 2 3 4
5	VNIR 2 (red)	71,8%	1 2 3 4 5
6	SAR September S6	67,4%	1 2 3 4 5 6
7	SAR September S2	61,9%	1 2 3 4 5 6 7
8	SWIR 3	59,5%	1 2 3 4 5 6 7 8
9	SWIR 2	59,1%	1 2 3 4 5 6 7 8 9
10 (least useful)	SWIR 5	56,6%	1 2 3 4 5 6 7 8 9 10

**Table 7.** Classification accuracy obtained from the different subset of features for the classification of vegetation types of the *veredas* and surroundings (P% represents the producer's accuracy and U% the user's).

**Samples**		**VNIR**		**VNIR and SWIR(3)**		**VNIR and SAR (April)**		**SAR (all 4)**	
**Class**	(n)	P%	U%	P%	U%	P%	U%	P%	U%

Grassland	48	77.1	64.9	68.8	57.9	68.8	62.3	62.5	30.9
Shrubland	60	21.7	56.5	23.3	60.9	15.0	69.2	6.7	5.8
Riparian forest	176	84.1	84.1	84.1	69.5	83.5	87.5	84.7	87.1
Savanna (sparse)	50	78.0	68.4	28.0	41.2	96.0	69.6	50.0	32.9
Savanna (dense)	180	87.8	81.9	67.2	77.1	88.9	83.3	39.4	54.2
Bare soil (sand)	42	90.5	76.0	90.5	52.8	92.9	57.4	4.8	9.1
Open water	23	100.0	100.0	100.0	100.0	69.6	100.0	56.5	100.0

Overall success		78.8		67.5		78.1		50.8	
Kappa (***k̂***)		75.2		58.2		71.8		42.8	

**Table 8.** Percentage of the vegetation cover captured by the unsupervised classification/labeling process of each of the four Radarsat-1 images (based on the ASTER VNIR classification and the 2,000 m buffer).

**Vegetation**	**April**		**September**	
	S2%	S6%	S2%	S6%
Grassland	38.2	30.8	31.6	17.7
Shrub	28.6	37.4	30.3	31.8
Riparian forest	76.4	77.7	78.0	64.7
Sparse savanna	11.3	11.4	9.8	9.2
				
Dense savanna	13.6	10.4	20.5	28.0
Bare soil	14.9	7.5	8.0	4.8
